# Safety and Efficacy of a Single Injection of Liposomal Latanoprost in Open-Angle and Angle-Closure Eyes

**DOI:** 10.3390/pharmaceutics18101223

**Published:** 2026-09-27

**Authors:** Shayne S. Tan, Phey Feng Lo, Ryan Eyn Kidd Man, Soh Chin Teo, Shamira A. Perera, Tina T. Wong

**Affiliations:** 1 Singapore National Eye Centre, 11 Third Hospital Avenue, Singapore 168751, Singapore; 2 Singapore Eye Research Institute, The Academia, 20 College Rd, Level 6 Discovery Tower, Singapore 169856, Singapore; 3 Ophthalmology and Visual Sciences Academic Clinical Programme, Duke-NUS Graduate Medical School, 8 College Rd, Singapore 169857, Singapore

**Keywords:** liposomes, nanomedicine, sustained drug delivery, glaucoma

## Abstract

**Background/Objectives:** Glaucoma is a leading cause of irreversible global vision loss, with intraocular pressure (IOP) as the sole modifiable risk factor to stall progression. First-line treatment in the form of eye drops suffers from poor bioavailability and low patient adherence, necessitating sustained-release alternatives. This study evaluates the safety and sustained efficacy of a liposomal latanoprost (LipoLat) injection in both open-angle and angle-closure eyes. **Methods:** This was a 3-month open-label, non-comparative pilot study that included patients aged 40 to 90 with ocular hypertension, primary open- and closed-angle glaucoma. After a 1-month washout from daily topical prostaglandin analogues, participants received a single sub-Tenon’s injection of LipoLat 2 mg/mL. Primary and secondary outcome measures included safety (the incidence of serious adverse events) and IOP-lowering efficacy. **Results:** Nine of 12 enrolled subjects completed the 3-month follow-up. LipoLat 2 mg/mL was safe, with no serious adverse events reported. There was a significant IOP reduction from a baseline of 23.1 ± 1.76 mmHg to 18.0 ± 1.42 mmHg at month 3 (*p* < 0.001), representing a maximum 22.6% decrease. Adjusted mixed models confirmed a 5.11 mmHg mean IOP reduction at 3 months, with five subjects showing sustained IOP reduction beyond 3 months, and two subjects remaining medication-free for up to 9 months. **Conclusions:** In this study, LipoLat is shown to be a safe, well-tolerated treatment for the first time in angle-closure eyes. Its sustained efficacy may also extend beyond 3 months. Larger randomised trials are warranted to further confirm the clinical application in angle-closure eyes.

## 1. Introduction

Glaucoma is a chronic, progressive optic neuropathy and the leading cause of irreversible visual loss globally as a result of the loss of retinal ganglion cells [1,2,3]. Glaucoma is projected to affect up to 111.8 million people worldwide by 2040 [2], of which primary angle-closure glaucoma (PACG) and primary open-angle glaucoma (POAG) are two of the most common subtypes. PACG accounts for nearly half of all primary glaucoma cases [3,4,5]; the global prevalence of PACG is expected to reach 32 million by 2040, with 76% of cases occurring in Asia [2,6]. POAG contributes to an estimated 53 million people worldwide as of 2020 and is projected to afflict up to 80 million people by 2040. Taken together, POAG and PACG contribute significantly to global visual burden, and yet, there is no cure. Intraocular pressure (IOP) remains the only modifiable risk factor in the treatment of glaucoma to halt or stall progression.

Whilst there have been significant advancements in IOP-lowering therapy for glaucoma, the mainstay first-line treatment modality for the majority of PACG or POAG patients is topical eye drops [7]. Prostaglandin analogues such as latanoprost have proven to be the most efficacious IOP-lowering topical medication amongst various classes, reducing IOP by approximately 25–35% when administered as once-daily eye drops [8]. Although such drugs are effective in reducing IOP, significant limitations to their efficacy exist, including human and anatomical factors, that result in poor drug bioavailability [7]. The conjunctival cul-de-sac has a maximum capacity of only 30 μL, and static barriers like the precorneal tear film and the tight junctions of the corneal epithelium, as well as dynamic barriers like reflex tearing and tear turnover time, put the bioavailability of eye drops at 5% [9]. However, the greatest challenge in glaucoma treatment lies in poor patient adherence, with studies showing up to 80% of patients failing to adhere to their prescribed anti-glaucoma treatment regimen [10]. Indeed, the use of more complex regimens (e.g., twice- or thrice-daily dosing) has been shown to further reduce patient adherence to eye drop instillation [11]. As such, long-term sustained release and delivery of IOP-lowering medication remains an attractive alternative to eye drops [7,12], mitigating the risks associated with poor drug bioavailability and patient adherence while avoiding the morbidity and risks from major surgical interventions such as trabeculectomy.

Our group has previously developed a liposomal latanoprost formulation at a drug/lipid mole ratio of 0.104 (latanoprost mass of 100 μg and liposome mass of 1.19 mg), and a mean particle size of approximately 100 μm [13]. Sustained and reproducible IOP lowering following a single subconjunctival injection was shown in New Zealand White rabbit eyes. Furthermore, sustained IOP lowering was achieved up to 120 days in ocular hypertensive Macaque monkeys after a single subconjunctival injection of liposomal latanoprost (1 mg/mL) [14]. A Phase I clinical trial in eyes with ocular hypertension and open-angle glaucoma showed that a single subconjunctival administration of liposomal latanoprost was safe and achieved IOP reduction up to 3 months post-injection [15]. However, to our knowledge, there are no studies thus far that report the use of a sustained drug delivery system for the management of angle-closure glaucoma. Furthermore, we also wanted to evaluate whether a higher concentration of liposomal latanoprost formulation of 2 mg/mL could have an improved sustained IOP-lowering effect.

In this study, we evaluated the safety and efficacy of LipoLat 2 mg/mL in both open-angle and angle-closure eyes.

## 2. Materials and Methods

This study was approved by the SingHealth Centralised Institutional Review Board (CIRB: 2024/2139) and adhered to the Declaration of Helsinki and Good Clinical Practice guidelines. This study was also approved by the Singapore Health Sciences Authority (HSA) (trial number CTA2400105). Written informed consent was obtained from all study participants.

This was an open-label, non-comparative pilot study evaluating LipoLat 2 mg/mL conducted over 3 months. There was a follow-up extension beyond 3 months for subjects who continued to have clinically significant IOP lowering, without the need to restart anti-glaucoma eye drops. We recruited patients aged 40 to 90 years, with diagnoses of OHT, PAC, or mild/moderate POAG, and PACG (mean deviation (MD) on visual field of ≤−12 dB). Angle closure was defined as the presence of closed angles (≥180° of the posterior pigmented trabecular meshwork not visible on non-indentation gonioscopy) [16]. Patients with OHT and PAC had no structural or functional evidence of glaucomatous optic neuropathy on optical coherence tomography (OCT) scans (Cirrus 4000; Carl Zeiss Meditec) or HVF (Carl Zeiss Meditec, Dublin, CA, USA). All patients were using a single prostaglandin analogue administered topically and were subjected to a washout period of 1 month, with post-washout unmedicated IOP of ≥22 mmHg as measured by a Goldmann Applanation Tonometer (GAT) and a best-corrected visual acuity (BCVA) of logMAR 1.0 or better in one or both eyes. Exclusion criteria were eyes with post-washout IOP of >30 mmHg, corneal thickness < 460 μm or >620 μm, secondary glaucoma, advanced glaucoma (mean deviation (MD) ≤ −12.0 dB), poorly controlled glaucoma (MD ≥ −12 dB), progressive glaucoma despite treatment, and previous laser procedures with the exception of peripheral laser iridotomy and/or laser trabeculoplasty ≥6 months ago. Eyes with previous intraocular glaucoma surgeries (e.g., minimally invasive glaucoma surgery (MIGS), trabeculectomies, etc.), documented non-response or contraindications to prostaglandin analogues, and patients who were using topical, nasal, inhaled, or systemic corticosteroids were also excluded. Patients were recruited only if they met all inclusion and exclusion criteria and proceeded to the baseline (day 0) visit.

### 2.1. Study Visits

At the baseline (day 0) visit, demographics and baseline ocular parameters were recorded for all patients, including BCVA on manifest refraction with ETDRS criteria on a backlit light box, IOP via GAT at 0900 h, 1200 h, and 1600 h. OCT scans of the retinal nerve fibre layer (RNFL) and an automated HVF 24-2 were taken within 6 months of the intervention.

### 2.2. Injection of LipoLat

At the start of the study, patients received a single subconjunctival injection of LipoLat 2 mg/mL under topical anaesthesia (proxymetacaine 0.5% eye drops) under aseptic conditions after the 1600 h IOP measurement. Prior to the procedure, povidone–iodine and one drop of phenylephrine 2.5% were instilled in the study eye to blanch the bulbar conjunctival vessels to decrease systemic distribution of the drug. A proxymetacaine-soaked cotton bud was applied to the intended injection site for 30 s to blanch any conjunctival blood vessels. Thereafter, 200 µL of LipoLat 2 mg/mL was delivered into the subconjunctival space at the superotemporal or inferotemporal bulbar conjunctiva via a 30G needle, followed by a proxymetacaine-soaked cotton bud applied to the injection site for 30 s to prevent drug reflux. This subconjunctival injection technique was subsequently modified to be a sub-Tenon’s technique after immediate post-injection needle track leak was noted in the first 3 patients (please see Results). One hour post-procedure, all eyes were re-examined under the slit lamp, and IOP was recorded. All patients were given a 5-day course of fortified levofloxacin 0.5% eye drops to be instilled 4 times a day.

Study visits were scheduled at 0900 h for the following visits: week 1, week 2, month 1, month 2, and month 3 post-operatively, where IOP was checked via GAT. In addition, the month 1 and month 3 visits also had diurnal IOP measurements at 1200 h and 1600 h. For patients who continued to have sustained IOP lowering, there was an extension of follow-up with the aforementioned procedures performed at month 4, month 6, month 9, and month 12. At each follow-up visit, any symptoms of ocular irritation, pain, blurring of vision, redness or discharge, foreign body sensation, and tearing were documented, along with BCVA and slit lamp examination to monitor for adverse reactions. These include examination findings such as conjunctival redness and integrity, corneal assessment (including fluorescein examination), iris and lens status, anterior chamber depth and activity, as well as the presence of subconjunctival haemorrhage, localised injection-site reactions, and infections. All subjects were also given a questionnaire to complete at the end of the study to collect feedback on their experience.

Topical anti-glaucoma medications were not introduced for at least 3 months after the procedure, unless the IOP was deemed by the investigator as posing an immediate risk to the study eye. This included IOP exceeding target thresholds (target IOP, pre-washout IOP, and post-washout IOP), signs of glaucomatous disease progression despite adequate IOP control, or patient request. The primary outcome measure was safety as determined by the incidence of serious adverse events (SAEs), with the secondary outcome measure being efficacy as measured by IOP lowering.

### 2.3. Statistical Analysis

All statistical analyses were performed using STATA 17.0 (Statacorp, College Station, TX, USA). Continuous variables were presented as mean ± standard deviation (SD), and categorical variables as counts and percentages. To assess the reduction in IOP 1 h post-injection, a paired *t*-test was run. To assess the reduction in IOP over study time points up to 6 months (24 weeks) compared to the post-washout IOP, mixed models employing restricted likelihood estimation were utilised to address the small sample size and potential intraocular correlation (model 1). Subsequently, these models were adjusted for potential confounding from age and gender (model 2). Paired t-tests were constructed to assess the differences in IOP between the baseline measurement at participant enrolment (prior to the initiation of wash-out) and each subsequent study time point up to 6 months. A two-sided *p*-value of less than 0.05 was deemed statistically significant.

## 3. Results

**Figure 1:**
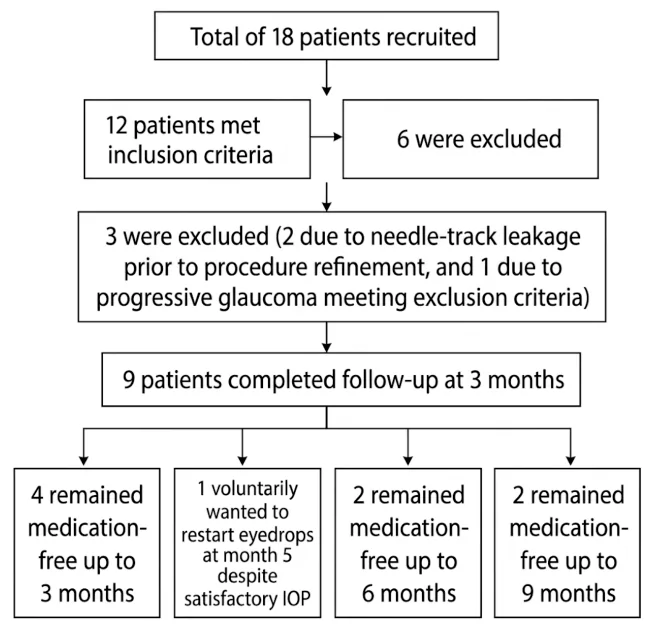
Flowchart of patients recruited and eventually included, as well as medication-free outcomes following sub-Tenon’s LipoLat injections.

Of 18 subjects screened, 12 met the eligibility criteria and were enrolled following washout (Figure 1). Nine of the 12 subjects enrolled completed all study visits and were analysed upon exit at 3 months. Baseline sociodemographic characteristics of the study population are shown in Table 1. There were seven male patients (58.3%), 10 (83.3%) were Chinese, and the mean age was 65 ± 8.7 years. In the study eye, eight had a diagnosis of open-angle glaucoma (OHT/POAG), and four had closed-angle (PAC/PACG) glaucoma. All subjects were using a prostaglandin analogue prior to washout (nine were using latanoprost and three travoprost).

Three patients were excluded from the final analysis as they exited the study early (Figure 1). Of these, two patients were the first two patients that were recruited by the study, and LipoLat was injected into the subconjunctival space. Immediate leakage of LipoLat through the needle track was observed by the attending ophthalmologist in both patients, and no significant bleb was noted in both eyes at 1 h post-injection. Due to the needle-track leak, the injection technique was modified to a sub-Tenon injection at 2 mm from the limbus, which requires longer tunnelling of the 30G needle into the subconjunctival space to reach the Tenon’s tissue, with tamponade with a cotton bud after injection, thereby limiting immediate needle-track leak. All subsequent patients recruited employed this technique of a sub-Tenon’s injection of LipoLat. The third excluded patient had a progressive rise in IOP from day 1 and required early resumption of topical anti-glaucoma medications at the month 2 visit when it was noticed that his IOP was higher than washout baseline IOP. In retrospect, this patient was found to be a non-responder to previous selective laser trabeculoplasty with resultant progressive glaucoma and required rapid eyedrop escalation to two eyedrop medications rather than just restarting latanoprost to achieve target low IOP. Of these three patients who were excluded, two had POAG, and one had PACG, leaving six open-angle patients and three angle-closure patients for analysis (n = 9) (Figure 1).

### 3.1. Safety

Three eyes had a mild subconjunctival haemorrhage immediately post-injection, which settled after a week. All patients reported no discomfort or pain following the injection, and all subjects maintained their best-corrected visual acuity (BCVA) throughout the study period. There were no SAEs throughout the follow-up period in all patients; in particular, there were no localised injection-site reactions or infections, corneal epithelial or endothelial loss of integrity, changes to the iris or lens status, or changes in anterior chamber depth and activity.

### 3.2. Efficacy

**Figure 2:**
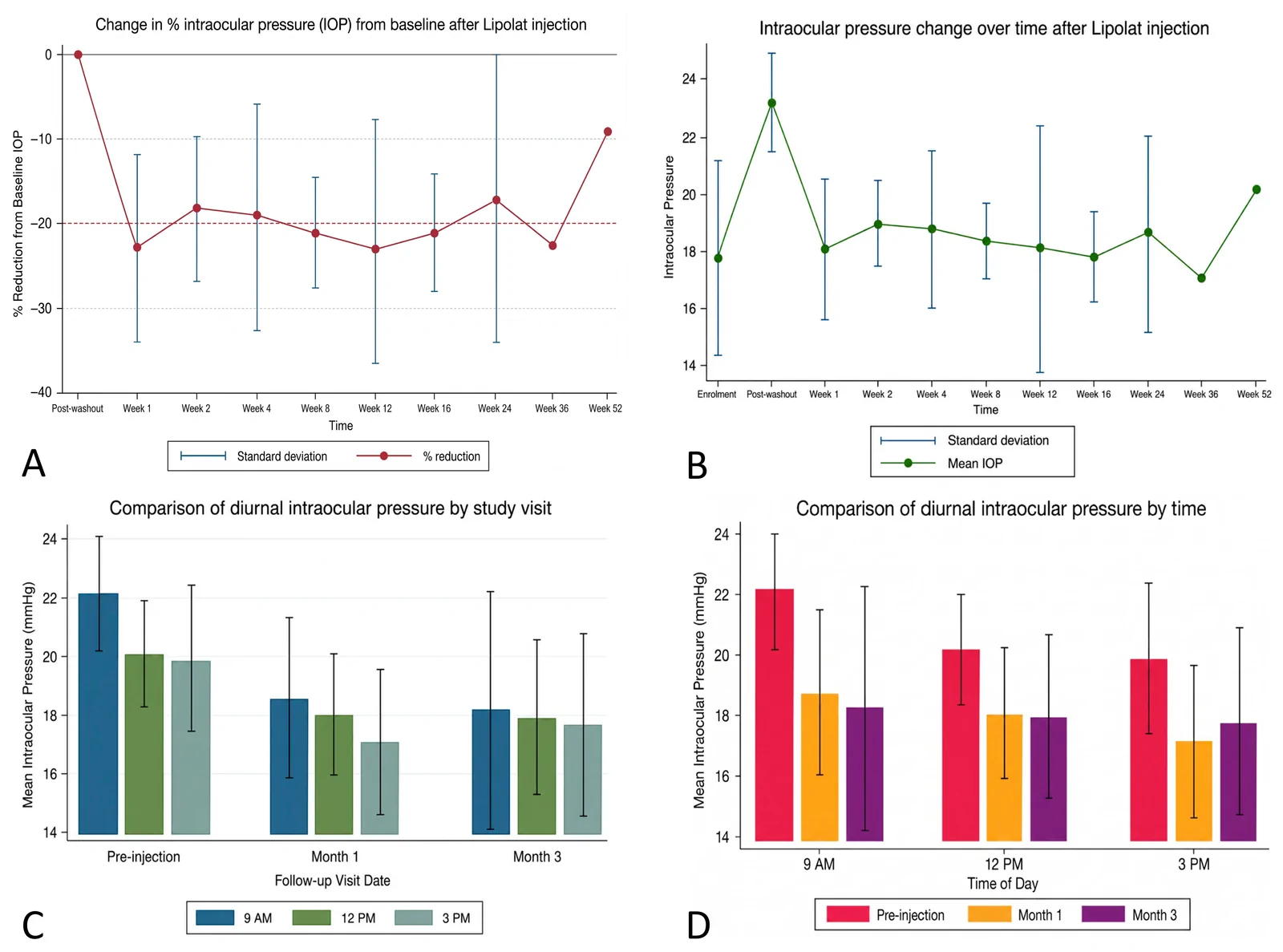
(**A**) Mean IOP change from baseline through to 12 weeks post-LipoLat injection. (**B**) Change (%) in intraocular pressure (IOP) from baseline through to 12 weeks post-LipoLat injection. (**C**) Change in diurnal IOP post-LipoLat injection at months 1 and 3. (**D**) Change in diurnal IOP by time of day post-LipoLat injection at months 1 and 3.

Of the nine patients that completed the 3-month study, mean IOP value pre-washout was 17.7 ± 3.35 mmHg, and post-washout was 23.1 ± 1.76 mmHg. At 1 h post-injection, all subjects demonstrated a decrease in IOP, with a mean IOP of 18.2 ± 3.42 mmHg, representing a mean decrease of 4.89 ± 2.37 mmHg (*p* < 0.01) and an IOP reduction of up to 21.5%. At week 1, month 1, and month 3, the mean IOP was 17.9 ± 2.42 mmHg, 18.7 ± 2.73 mmHg, and 18.0 ± 1.42 mmHg, respectively (Figure 2A), representing a mean sustained decrease of up to 22.6% from baseline (Figure 2B). Mean decreases at each of these study time points were statistically significant, *p* < 0.001. Mixed models adjusted for age and gender showed that a single sub-Tenon’s LipoLat injection maintained a mean IOP reduction of 5.11 mmHg (3.14, 7.07, 95% CI) at month 3, 4.27 mmHg (1.40, 7.13, 95% CI) at month 4, and 3.72 mmHg (1.17, 6.28, 95% CI) at month 6 post-injection (Table 2).

Diurnal phasing of IOP also showed a significant sustained decrease in IOP across all time points throughout the day through to month 3 (all *p* < 0.01, Figure 2C,D). Interestingly, sustained IOP lowering was observed beyond month 3. Of the nine subjects analysed, four remained medication-free up to month 3, and five showed IOP lowering beyond 3 months, of whom three were open-angle and two were angle-closure patients (Table 3). Of the five subjects that had sustained IOP lowering beyond 3 months, one had IOP-lowering up to 5 months, and two up to 6 months. The last two patients remained medication-free through to month 9, maintaining a mean IOP reduction of 26.6% (Figure 1, Table 3). The subject who had topical glaucoma medications restarted at month 5 had the resumption carried out at the patient’s request despite adequate IOP control of 19 mmHg; this subject’s post-washout IOP was 23 mmHg.

A sub-analysis of IOP reduction between open versus closed angle groups showed mean IOP reduction of 5.83 ± 3.54 mmHg (*p* < 0.01) versus 3.67 ± 2.52 mmHg at month 3 post-injection, respectively. The *p*-value was not significant for the closed-angle group due to the small sample size of the group (three subjects). Despite that, two of the three angle-closure subjects reported sustained IOP lowering beyond month 3, with glaucoma medications restarted only at month 6.

All subjects reported a positive experience during the study, noting that the injection was comfortable and well tolerated, and all participants indicated that they would recommend the treatment to other fellow patients with similar clinical conditions. Moreover, the subjects were all willing to switch from their daily eye drop regimen to a 3-monthly sub-Tenon’s injection of LipoLat.

## 4. Discussion

In this pilot study, we demonstrated the efficacy of LipoLat at a concentration of 2 mg/mL to be safe and able to lower IOP in both open- and closed-angle glaucoma. IOP lowering achieved a 22.6% reduction from washout IOP through to 3 months post-injection, with 56% of subjects remaining off eyedrops beyond 3 months following a single sub-Tenon’s injection. The modification to a sub-Tenon’s injection was deemed to be safe, with good tolerability of the procedure reported in all subjects.

Of note, this is the first clinical study that investigates the use of a sub-Tenon’s injected drug delivery system to achieve sustained IOP lowering, especially in angle-closure patients. The only two FDA-approved drug delivery systems in the market for glaucoma patients are Durysta and iDose-TR, which are intracameral trabecular meshwork-based implants of bimatoprost and travaprost, respectively, designed to produce sustained IOP-lowering effects of at least 3 months in glaucoma patients with open angles [7]. However, their use is contraindicated in patients with angle closure, as the trabecular meshwork may not be well visualised [17,18]. In this respect, LipoLat, a liquid nanoliposomal formulation, shows advantages that can overcome the safety concerns of delivery systems placed in the anterior chamber. As reported in this study, we report the clinical utility of LipoLat in lowering IOP in angle-closure eyes with a clinically relevant trend of IOP reduction (3.67 ± 2.52 mmHg). Furthermore, two of the three angle-closure patients had sustained IOP lowering beyond 3 months, up to 6 months post-injection. However, this study remains a pilot study that evaluates safety with only a small number of angle-closure patients, but it can pave the way for a subsequent larger clinical trial to establish clinical efficacy.

Patient adherence has always been one of the biggest challenges in the management of glaucoma, with studies showing that up to 50% of glaucoma patients were non-adherent to their prescribed medication regimen over 75% of the time [19,20]. Studies have shown that up to 75% of glaucoma patients were willing to accept alternative sustained ocular drug delivery systems [21,22,23] such as 3-monthly subconjunctival injections rather than daily eyedrop use. A total of 86% of these patients were willing to pay for this alternative treatment even at a higher cost [22] and up to 53% accepted the use of subconjunctival injections as a treatment alternative if they were equal or more effective than eye drops [24]. Similarly, in our study, patient feedback was highly positive, with all patients reporting that the LipoLat injection was well-tolerated and that they were comfortable throughout the procedure. All patients were willing to switch from daily eye drop use to 3-monthly injections, and all patients were also willing to pay more for a single injection of LipoLat than what they would otherwise have paid per month.

Our group had previously studied the safety and efficacy of LipoLat 1 mg/mL in a pilot study on six OHT and POAG patients, finding a clinically and statistically significant IOP reduction of about 20% through 3 months after a single subconjunctival injection [15]. In this study, we also showed that LipoLat 2 mg/mL not only demonstrated a similar IOP reduction of up to 22.6% at 3 months post-injection as with the lower concentration [25], but that the IOP-lowering efficacy is comparable to the IOP reduction with daily eyedrop instillation of latanoprost. In addition, this study observed four subjects remaining drop-free up to 6 months, of which two remained drop-free up to 9 months. These long-term IOP-lowering effects of prostaglandin analogues have been well described in animal models of Durysta (an FDA-approved intracameral bimatoprost), which showed that even after all of the bimatoprost had been released at 90 days, IOP-lowering effects persisted up to 1–2 years after a single administration of Durysta [26,27,28]. However, the mechanism of such longer-term IOP-lowering effects is not fully understood, with some studies suggesting that this involves upregulation of matrix metalloproteinase 1 (MMP1) and downregulation of fibronectin, hence promoting sustained tissue remodelling of aqueous outflow in the trabecular meshwork [29]. Therefore, it could be further hypothesised that the IOP-lowering effect would extend for longer periods and that the time interval between injections may be longer after a few injections. More studies are required to establish the clinical efficacy of LipoLat with larger randomised controlled trials and control groups, to understand the mechanism of sustained action following a relatively small volume injected, and also to establish the pharmacokinetic biodistribution of the LipoLat post-injection over several months.

The relative ease and safety profile of a sub-Tenon’s injection makes LipoLat a potential nurse-led in-office procedure in the future, expanding the scope of healthcare professionals and reducing in-office waiting time for patients. Nurse-led ophthalmology procedures are not new, and in fact, intravitreal injections (IVTs) of anti-vascular endothelial growth factor (VEGF), which were primarily performed by ophthalmologists, are now almost universally performed by nurses without compromising safety or efficacy [30,31,32]. Sub-Tenon’s injection of LipoLat has a relatively easy learning curve and is procedurally simple, with much lower risks compared to IVTs, portending good scalability of LipoLat injections to reduce patient wait times and increase patient acceptability. However, based on the results from this study, we advocate performing the injections in a modified way into the sub-Tenon’s space as described above in order to minimise drug loss with a small injected volume, and achieve a more consistent performance from the drug. Integrating a three-monthly LipoLat sub-Tenon’s injection regimen into existing ophthalmic workflows can effectively mirror the highly successful, nurse-led models currently established for IVTs, such as in the treatment of diabetic macular oedema and age-related macular degeneration. Under a similar parallel framework, stable glaucoma patients could be transitioned to a dedicated nurse-led injector clinic where trained ophthalmic nurses independently conduct IOP checks and administer LipoLat. This task-shifting strategy significantly optimises clinic time for stable patients, effectively reserving ophthalmologist slots for complex or unstable cases.

Our study has several limitations. First, this was a pilot study, with a small sample size, and investigators and subjects were not masked. While results are promising, larger blinded randomised controlled studies with more patients, especially those with angle closure, should be conducted to confirm the sustained IOP reduction. Secondly, there was also no control group to provide a comparison in efficacy against topical latanoprost ophthalmic solutions, especially the regression to the mean. However, future larger clinical trials will help to address these limitations. As nine out of 12 patients were on latanoprost, there may be bias, as we used a population in which we knew LipoLat would work. Finally, treatment-naïve patients were not recruited, which may limit generalizability to real-world treatment-naïve populations.

In conclusion, this study is the first to evaluate a sustained delivery system in angle-closure eyes. LipoLat was shown to be safe in open and closed angle-closure eyes and demonstrated IOP lowering beyond 3 months in both groups following a single injection in a small pilot study. Future larger studies are warranted to confirm these preliminary findings.

## Figures and Tables

**Table 1:** Baseline demographics of all LipoLat study subjects.

Characteristics	Value
Total no. of patients (%)	12 (100%)
Male, number (%)	7 (58.3%)
Age, mean years ± SD	65.08 ± 9.40
Pre-washout IOP, mean (mmHg) ± SD	17.66 ± 3.35
Previous topical glaucoma medication, number (%)	
Latanoprost	9 (75.0%)
Travoprost	3 (25.0%)
Ethnicity, number (%)	
Chinese	10 (83.3%)
Indian	2 (16.7%)
Malay	0 (0%)
Others	0 (0%)
Diagnosis, number (%)	
Open angle (OHT/POAG)	8 (66.7%)
Closed angle (PAC/PACG)	4 (33.3%)
Prior cataract surgery, number (%)	3 (25.0%)
Prior laser treatment (LPI/SLT), number (%)	6 (50.0%)

SD: standard deviation; OHT: ocular hypertension; POAG: primary open-angle glaucoma; PAC: primary angle closure; PACG: primary angle-closure glaucoma; LPI: laser peripheral iridotomy; SLT: selective laser trabeculoplasty.

**Table 2:** Clinical effectiveness of LipoLat in reducing intraocular pressure over time.

Time	IOP (mmHg)	
	Model 1 (β [95% CI])	Model 2 (β [95% CI])
Post wash-out (n = 9); Reference group	23.11 (21.42, 24.79)	23.01 (21.51, 24.52)
Week 1 (n = 9)	−5.15 (−7.12, −3.20)	−5.16 (−7.13, −3.20)
Week 2 (n = 9)	−4.27 (−6.23, −2.31)	−4.27 (−6.24, −2.31)
Week 4 (n = 9)	−4.44 (−6.40, −2.48)	−4.44 (−6.40, −2.48)
Week 8 (n = 9)	−4.88 (−6.84, −2.92)	−4.88 (−6.85, −2.92)
Week 12 (n = 9)	−5.11 (−7.07, −3.15)	−5.11 (−7.07, −3.14)
Week 24 (n = 4)	−3.83 (−6.39, −1.28)	−3.72 (−6.28, −1.17)

Model 1: mixed models with restricted maximum likelihood estimation. Model 2: mixed models with restricted likelihood estimation, adjusting for age and gender.

**Table 3:** Sustained IOP lowering beyond month 3.

Subject	Diagnosis	IOP				
		Screening	Post washout	Month 3	Month 6	Month 9
1	OHT	17	22	18	17	17
2	PACG	18	22	16	19 *	
3	PAC	14	22	18	23 *	
4	OHT	14	23	12 **		
5	POAG	16	23	14	15	16
6	PAC	17	25	24 *		
7	OHT	17	22	20 *		
8	POAG	18	27	25 *		
9	POAG	18	22	21 *		

* glaucoma drops started. ** restarted topical therapy at month 5; IOP 19 due to patient choice.

## Data Availability

Our raw data cannot be shared openly in order to protect the privacy of the participants. Data would be partially available from the corresponding authors S.A.P. and T.T.W., upon reasonable request.

## References

[1] Steinmetz J.D., Bourne R.R.A., Briant P.S., Flaxman S.R., Taylor H.R.B., Jonas J.B., Abdoli A.A., Abrha W.A., Abualhasan A., Abu-Gharbieh E.G. (2021). Causes of Blindness and Vision Impairment in 2020 and Trends over 30 Years, and Prevalence of Avoidable Blindness in Relation to VISION 2020: The Right to Sight: An Analysis for the Global Burden of Disease Study. Lancet Glob. Health.

[2] Tham Y.-C., Li X., Wong T.Y., Quigley H.A., Aung T., Cheng C.-Y. (2014). Global Prevalence of Glaucoma and Projections of Glaucoma Burden through 2040. Ophthalmology.

[3] Quigley H.A. (2006). The Number of People with Glaucoma Worldwide in 2010 and 2020. Br. J. Ophthalmol..

[4] Tan S.S., Tun T.A., Aung T., Nongpiur M.E. (2024). Comparison of Intraocular Pressure and Anterior Segment Parameters in Subjects with Asymmetrical Primary Angle Closure Disease. Clin. Exp. Ophthalmol..

[5] George R., Panda S., Vijaya L. (2022). Blindness in Glaucoma: Primary Open-Angle Glaucoma versus Primary Angle-Closure Glaucoma-a Meta-Analysis. Eye.

[6] Alsagoff Z., Aung T., Ang L.P., Chew P.T. (2000). Long-Term Clinical Course of Primary Angle-Closure Glaucoma in an Asian Population. Ophthalmology.

[7] Tan S.S., Wong T.T. (2025). Drug Delivery Systems in Glaucoma—Current Innovations and Future Perspectives. Asia-Pac. J. Ophthalmol..

[8] Larsson L.I. (2001). Intraocular Pressure over 24 Hours after Single-Dose Administration of Latanoprost 0.005% in Healthy Volunteers. A Randomized, Double-Masked, Placebo Controlled, Cross-over Single Center Study. Acta Ophthalmol. Scand..

[9] Agrahari V., Mandal A., Agrahari V., Trinh H.M., Joseph M., Ray A., Hadji H., Mitra R., Pal D., Mitra A.K. (2016). A Comprehensive Insight on Ocular Pharmacokinetics. Drug Deliv. Transl. Res..

[10] Olthoff C.M.G., Schouten J.S.A.G., van de Borne B.W., Webers C.A.B. (2005). Noncompliance with Ocular Hypotensive Treatment in Patients with Glaucoma or Ocular Hypertension an Evidence-Based Review. Ophthalmology.

[11] Robin A.L., Novack G.D., Covert D.W., Crockett R.S., Marcic T.S. (2007). Adherence in Glaucoma: Objective Measurements of Once-Daily and Adjunctive Medication Use. Am. J. Ophthalmol..

[12] So Y.H., Mishra D., Gite S., Sonawane R., Waite D., Shaikh R., Vora L.K., Thakur R.R.S. (2025). Emerging Trends in Long-Acting Sustained Drug Delivery for Glaucoma Management. Drug Deliv. Transl. Res..

[13] Natarajan J.V., Chattopadhyay S., Ang M., Darwitan A., Foo S., Zhen M., Koo M., Wong T.T., Venkatraman S.S. (2011). Sustained Release of an Anti-Glaucoma Drug: Demonstration of Efficacy of a Liposomal Formulation in the Rabbit Eye. PLoS ONE.

[14] Natarajan J.V., Darwitan A., Barathi V.A., Ang M., Htoon H.M., Boey F., Tam K.C., Wong T.T., Venkatraman S.S. (2014). Sustained Drug Release in Nanomedicine: A Long-Acting Nanocarrier-Based Formulation for Glaucoma. ACS Nano.

[15] Wong T.T., Novack G.D., Natarajan J.V., Ho C.L., Htoon H.M., Venkatraman S.S. (2014). Nanomedicine for Glaucoma: Sustained Release Latanoprost Offers a New Therapeutic Option with Substantial Benefits over Eyedrops. Drug Deliv. Transl. Res..

[16] Foster P.J. (2002). The Definition and Classification of Glaucoma in Prevalence Surveys. Br. J. Ophthalmol..

[17] Glaukos (2023). Prescribing Information for iDose TR.

[18] AbbVie Inc. (2024). Prescribing Information for Durysta® (Bimatoprost Intracameral Implant) for Intracameral Administration.

[19] Okeke C.O., Quigley H.A., Jampel H.D., Ying G., Plyler R.J., Jiang Y., Friedman D.S. (2009). Interventions Improve Poor Adherence with Once Daily Glaucoma Medications in Electronically Monitored Patients. Ophthalmology.

[20] Robin A., Grover D.S. (2011). Compliance and Adherence in Glaucoma Management. Indian J. Ophthalmol..

[21] Weber C., Quintin P., Holz F.G., Fea A., Mercieca K. (2024). Ocular Drug Delivery Systems: Glaucoma Patient Perceptions from a German University Hospital Eye Clinic. Graefes Arch. Clin. Exp. Ophthalmol..

[22] Chong R.S., Su D.H.W., Tsai A., Jiang Y., Htoon H.M., Lamoureux E.L., Aung T., Wong T.T. (2013). Patient Acceptance and Attitude Toward an Alternative Method of Subconjunctival Injection for the Medical Treatment of Glaucoma. J. Glaucoma.

[23] Chan H.H., Wong T.T., Lamoureux E., Perera S. (2015). A Survey on the Preference of Sustained Glaucoma Drug Delivery Systems by Singaporean Chinese Patients: A Comparison Between Subconjunctival, Intracameral, and Punctal Plug Routes. J. Glaucoma.

[24] Varadaraj V., Kahook M.Y., Ramulu P.Y., Pitha I.F. (2018). Patient Acceptance of Sustained Glaucoma Treatment Strategies. J. Glaucoma.

[25] Zhang W.Y., Po A.L., Dua H.S., Azuara-Blanco A. (2001). Meta-Analysis of Randomised Controlled Trials Comparing Latanoprost with Timolol in the Treatment of Patients with Open Angle Glaucoma or Ocular Hypertension. Br. J. Ophthalmol..

[26] Craven E.R., Walters T., Christie W.C., Day D.G., Lewis R.A., Goodkin M.L., Chen M., Wangsadipura V., Robinson M.R., Bejanian M. (2020). 24-Month Phase I/II Clinical Trial of Bimatoprost Sustained-Release Implant (Bimatoprost SR) in Glaucoma Patients. Drugs.

[27] Medeiros F.A., Walters T.R., Kolko M., Coote M., Bejanian M., Goodkin M.L., Guo Q., Zhang J., Robinson M.R., Weinreb R.N. (2020). Phase 3, Randomized, 20-Month Study of Bimatoprost Implant in Open-Angle Glaucoma and Ocular Hypertension (ARTEMIS 1). Ophthalmology.

[28] Bacharach J., Tatham A., Ferguson G., Belalcázar S., Thieme H., Goodkin M.L., Chen M.Y., Guo Q., Liu J., Robinson M.R. (2021). Phase 3, Randomized, 20-Month Study of the Efficacy and Safety of Bimatoprost Implant in Patients with Open-Angle Glaucoma and Ocular Hypertension (ARTEMIS 2). Drugs.

[29] Stamer W.D., Perkumas K.M., Kang M.H., Dibas M., Robinson M.R., Rhee D.J. (2023). Proposed Mechanism of Long-Term Intraocular Pressure Lowering with the Bimatoprost Implant. Investig. Ophthalmol. Vis. Sci..

[30] Teo A.W.J., Rim T.H., Wong C.W., Tsai A.S.H., Loh N., Jayabaskar T., Wong T.Y., Cheung C.M.G., Yeo I.Y.S. (2020). Design, Implementation, and Evaluation of a Nurse-Led Intravitreal Injection Programme for Retinal Diseases in Singapore. Eye.

[31] Gregg E. Nurse-Led Ranibizumab Intravitreal Injections in Wet Age-Related Macular Degeneration: A Literature Review. https://journals.rcni.com/nursing-standard/nurseled-ranibizumab-intravitreal-injections-in-wet-agerelated-macular-degeneration-a-literature-review-ns.2017.e10344.

[32] DaCosta J., Hamilton R., Nago J., Mapani A., Kennedy E., Luckett T., Pavesio C., Flanagan D. (2014). Implementation of a Nurse-Delivered Intravitreal Injection Service. Eye.

